# Identification of exosome-like nanoparticle-derived microRNAs from 11 edible fruits and vegetables

**DOI:** 10.7717/peerj.5186

**Published:** 2018-07-31

**Authors:** Juan Xiao, Siyuan Feng, Xun Wang, Keren Long, Yi Luo, Yuhao Wang, Jideng Ma, Qianzi Tang, Long Jin, Xuewei Li, Mingzhou Li

**Affiliations:** 1 Sichuan Agricultural University, Institute of Animal Genetics and Breeding, College of Animal Science and Technology, Chengdu, People’s Republic of China; 2 Sichuan Agricultural University, Farm Animal Genetic Resources Exploration and Innovation Key Laboratory of Sichuan Province, Chengdu, People’s Republic of China

**Keywords:** Exosome-like nanoparticles, Cross-kingdom, miRNAs expression profile, Illumina sequencing, miRNA expression profile

## Abstract

Edible plant-derived exosome-like nanoparticles (EPDELNs) are novel naturally occurring plant ultrastructures that are structurally similar to exosomes. Many EPDELNs have anti-inflammatory properties. MicroRNAs (miRNAs) play a critical role in mediating physiological and pathological processes in animals and plants. Although miRNAs can be selectively encapsulated in extracellular vesicles, little is known about their expression and function in EPDELNs. In this study, we isolated nanovesicles from 11 edible fruits and vegetables and subjected the corresponding EPDELN small RNA libraries to Illumina sequencing. We identified a total of 418 miRNAs—32 to 127 per species—from the 11 EPDELN samples. Target prediction and functional analyses revealed that highly expressed miRNAs were closely associated with the inflammatory response and cancer-related pathways. The 418 miRNAs could be divided into three classes according to their EPDELN distributions: 26 “frequent” miRNAs (FMs), 39 “moderately present” miRNAs (MPMs), and 353 “rare” miRNAs (RMs). FMs were represented by fewer miRNA species than RMs but had a significantly higher cumulative expression level. Taken together, our *in vitro* results indicate that miRNAs in EPDELNs have the potential to regulate human mRNA.

## Introduction

Exosomes are small (30–100 nm) membranous nanovesicles secreted by a variety of mammalian cells (Bang & Thum, 2012; Denzer et al., 2000). These mRNA- and microRNA-rich microvesicles can be transferred into neighboring or distant cells and play an important role in intercellular communication (Redis et al., 2012; Théry, Zitvogel & Amigorena, 2002; Valadi et al., 2007). In recent years, plant-derived exosome-like nanoparticles (ELNs) have also been characterized and shown to have structures similar to those of mammalian exosomes (Mu et al., 2014; Zhang et al., 2016). Additionally, accumulating evidence suggests that edible plant-derived ELNs (EPDELNs) can be absorbed in the mammalian gastrointestinal (GI) tract and have the potential to mediate plant–animal intercellular communication (Ju et al., 2013; Mu et al., 2014; Raimondo et al., 2015; Wang et al., 2014; Wang et al., 2015; Zhuang et al., 2015). For example, nanoparticles from four edible plants (grape, grapefruit, ginger, and carrot) have anti-inflammatory properties and help maintain intestinal homeostasis (Mu et al., 2014). Furthermore, ginger-derived nanoparticles can protect against the development of liver-related diseases such as alcohol-induced damage (Zhuang et al., 2015). Moreover, Ju et al. (2013) have demonstrated that grape exosome-like nanoparticles can be assimilated by mouse intestinal stem cells and promote intestinal stem cell proliferation through the Wnt/*β*-catenin pathway.

MicroRNAs (miRNAs) are a class of small (18–24 nt) noncoding RNAs that play important roles in post-transcriptional gene regulation in animals and plants (Bartel, 2004; Chen, 2012). These single-stranded molecules impact on fundamental biological processes, including proliferation, differentiation, immune responses, and metabolism (Bartel, 2004; Khan et al., 2011; Luo, Guo & Li, 2013; Waldron & Newbury, 2012; Xiao & Rajewsky, 2009). The abnormal expression of miRNAs is related to multiple diseases (Calin & Croce, 2006; Lu et al., 2005). Zhang et al. (2012a) were the first to report that plant-derived miRNAs can penetrate the mammalian GI tract and enter the serum. They also found that plant-derived MIR-168a can bind to the mRNA of low-density-lipoprotein receptor adapter protein 1 (LDLRAP1) to inhibit its expression in the liver and decrease LDL removal. However, controversy exists regarding cross-kingdom regulation by plant miRNAs (Dickinson et al., 2013; Snow et al., 2013), with some researchers suggesting that their existence in animal blood and tissues reflects sample contamination or nonfunctional ingestion (Snow et al., 2013; Zhang et al., 2012b). Nevertheless, subsequent studies support the view that diet-derived plant miRNAs are present in blood (Liang et al., 2015; Luo et al., 2017) and tissue (Luo et al., 2017) and can regulate endogenous gene expression in animals (Zhu et al., 2017).

Our previous study showed that maize-derived miRNAs can exist in the bloodstream and solid tissues of pigs, and can potentially target porcine mRNAs (Luo et al., 2017). Intriguingly, Zhu et al. (2017) recently reported a previously uncharacterized function of plant miRNAs in the fine-tuning of honeybee caste development. Although these results suggest that plant miRNAs can mediate cross-kingdom regulation, miRNAs encapsulated in EPDELNs remain to be identified. Therefore, in this study, EPDELNs were isolated from 11 edible plants, and their miRNAs were subjected to expression profiling by small-RNA sequencing. To reveal the potential roles of miRNAs in EPDELNs, we used *in silico* analysis and a dual-luciferase reporter assay to predict and validate relationships between EPDELNs miRNAs and their potential target genes. Our results showed that miRNAs enriched in EPDELNs have the potential to mediate interspecies intercellular communication.

## Materials and Methods

### Isolation and purification of ELNs

Eleven edible plants (blueberry, coconut, ginger, grapefruit, Hami melon, kiwifruit, orange, pea, pear, soybean, and tomato) were randomly chosen for this study. All EPDELN samples were isolated and purified by differential centrifugation as described previously (Ju et al., 2013; Mu et al., 2014; Wang et al., 2015; Zhuang et al., 2015). Fruits and vegetables were purchased from a local market and washed three times. Apart from coconut, juice was extracted in two different ways. Fruits and vegetables with abundant juice (blueberry, grapefruit, kiwifruit, orange, and pear) were peeled, wrapped in gauze, and squeezed by hand, while less juicy ones (ginger, Hami melon, pea, soybean, and tomato) were ground with phosphate-buffered solution (PBS) in a mixer. The collected juice was sequentially centrifuged at 1,200 × *g* for 20 min, 3,000 × *g* for 20 min, and 10,000 × *g* for 60 min at 4 °C in a Sorvall Lynx 6000 centrifuge (Fisher Scientific, Shanghai, China) to remove large particles and cellular debris. The supernatant was filtered through a 1-μm membrane filter (Millipore, Bedford, MA, USA) and then centrifuged at 150,000  × *g* for 90 min at 4 °C in an LE-80 ultracentrifuge (Beckman Coulter, Palo Alto, CA, USA) to obtain EPDELNs. EPDELNs were resuspended in 250 μl PBS.

### Atomic force microscopy (AFM) and nanovesicle particle size analysis

To morphologically characterize the isolated membrane fraction, specimens were diluted 1:1,000 in PBS, and absorbed onto freshly cleaved mica sheets for 20 min. To provide a surface coated with formulations to a suitable density, the mica sheets were rinsed three times with deionized water and then dried with filter paper before detection. Surface morphology was examined under an atomic force microscope (Asylum Research MFP-3D-Bio; Digital Instruments, Santa Barbara, CA, USA) as described by Mu et al. (2014). The particle size distribution of EPDELNs was evaluated using a Light Scattering System (Brookhaven BI-200SM) as previously described (Mu et al., 2014). Measurements were made in PBS at pH 7.0 at 25 °C after appropriate dilution of each EPDELN sample.

### Small RNA sequencing and data analysis

Total RNAs from the 11 EPDELN samples were separately obtained using TRIzol LS reagent (Invitrogen, Waltham, MA, USA) according to the manufacturer’s instructions. After resuspending each RNA sample in 30 μl RNase-free water (Takara Bio, Shiga, Japan), the RNA quality was examined by 1% agarose gel electrophoresis and on an Agilent 2100 Bioanalyzer (Agilent Technologies, Santa Clara, CA, USA). Samples were stored at −80 °C. To construct each library, small RNA ranging in size from 14 to 36 nt was purified by polyacrylamide gel electrophoresis and ligated using proprietary adaptors. The modified small RNA was then reverse-transcribed into cDNA and amplified by PCR. Finally, the 11 libraries were sequenced on an Illumina HiSeq 2500 platform.

The resulting sequence reads were subjected to a series of stringent filters, including the removal of low-quality reads, repeat sequences, and adaptor sequences, to generate clean data. A bioinformatics pipeline (MIRPIPE) for miRNA discovery and profiling was then applied as previously described (Kuenne et al., 2014). MIRPIPE was also applied in a subsequent analysis. In brief, the filtered reads were identified against known plant mature miRNAs in miRBase21.0 (November 2016; http://www.mirbase.org/index.shtml) using the criterion of a maximum of two mismatches. The identified reads were treated as conserved miRNAs, and the number of reads per miRNA were normalized to reads per million (RPM). The miRNA profiling data have been deposited in NCBI Gene Expression Omnibus (GEO: GSE116095) and are accessible through the GEO Super Series.

### Prediction and functional annotation of miRNA target genes

TargetScan (http://www.targetscan.org/vert_71/) was used to annotate miRNA target gene (Wei et al., 2017). The generated list of target genes was subsequently uploaded to the Database for Annotation, Visualization and Integrated Discovery (DAVID) bioinformatics resource (Huang da, Sherman & Lempicki, 2009) (https://david.ncifcrf.gov/) for Gene Ontology (GO) and Kyoto Encyclopedia of Genes and Genomes (KEGG) pathway analyses. DAVID GO enrichment was regarded as significant using a criterion of *P* < 0.05. Sequence information on human mRNAs was collected from the NCBI database (http://www.ncbi.nlm.nih.gov/). The NCBI database and RNAhybrid (https://bibiserv.cebitec.uni-bielefeld.de/rnahybrid/) were used in combination to search for human mRNAs containing potential binding sites for plant miRNAs.

### Cells, reagents, and oligonucleotides

The HeLa cell line obtained from the Chinese Academy of Sciences cell bank (SGST.CN) was cultured in Dulbecco’s modified Eagle’s medium (DMEM, Gibco, Waltham, MA, USA) supplemented with 10% fetal bovine serum (Gibco) and incubated at 37 °C in a humidified atmosphere containing 5% CO_2_. Synthetic miRNA molecules with 2′-O methylation at the 3′ end were purchased from Gene Pharma (Shanghai, China).

### Dual-luciferase reporter assay

To experimentally validate miRNA–target interactions, a dual-luciferase reporter assay system was used. Wild-type (WT) or mutant (MUT) binding sites were cloned into a pmirGLO vector (100 ng per well). The vector harboring WT or MUT binding sites combined with either synthetic MIR-168c (50 nM), synthetic MIR-8155 (50 nM), or an miRNA control (50 nM) were then co-transfected into HeLa cells grown in 96-well plates at a density of 5  × 10^3^ cells/well using Lipofectamine 3000 (0.3 μL per well) (Invitrogen, Waltham, MA, USA). Cells were collected after 48 h, and Renilla and firefly fluorescence levels were assayed using the Dual-Luciferase Reporter Assay System kit (Promega, Madison, WI, USA).

### Statistical analysis

Data were presented as means ± SEM. Statistical analyses were performed using the Student’s *t*-test, with differences considered significant at *p* < 0.05.

## Results

### Isolation and characterization of EPDELNs

**Figure 1:**
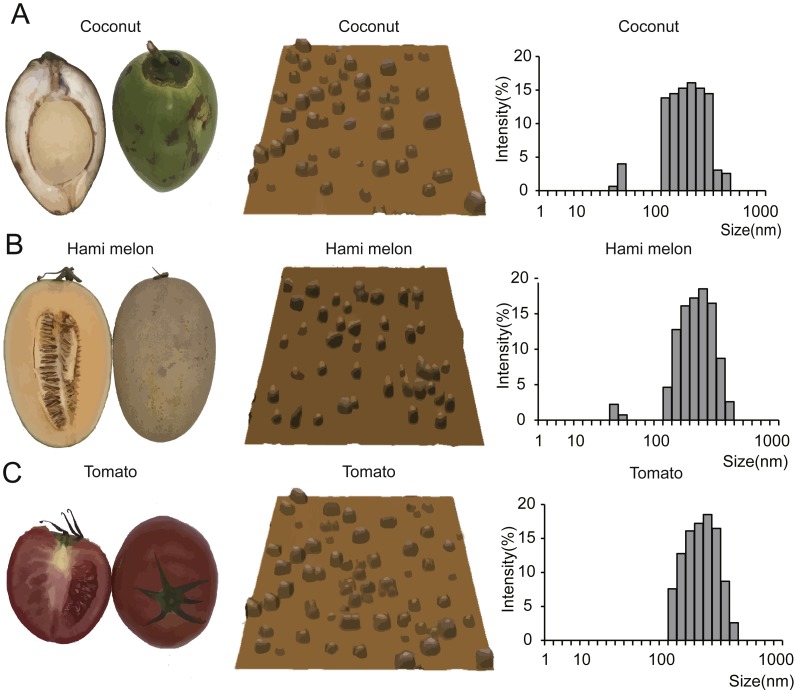
EPDELN morphological ultrastructure and size distribution. The morphological ultrastructure was visualized by AFM, and the size distribution of EPDELNs was analyzed by DLS in coconut (A), Hami melon (B), and tomato (C). Photographs by Juan Xiao.

To verify our hypothesis that miRNAs are enriched in EPDELNs and can potentially mediate interspecies intercellular communication, we randomly selected 11 commonly consumed fresh fruits and vegetables (Fig. 1 and Fig. S1). EPDELNs were isolated by differential centrifugation (Mu et al., 2014; Wang et al., 2013; Zhuang et al., 2015) and identified at the nanometer scale by AFM. The 11 different edible fruits and vegetables were found to contain a substantial quantity of round or oval vesicles (Fig. 1 and Fig. S1). These vesicles exhibited a morphological ultrastructure similar to that of exosomes from mammalian bodily fluids (Zhuang et al., 2015). To further characterize the EPDELNs, a dynamic light scattering (DLS) analysis was also performed. As shown in Fig. 1 and Fig. S1, the size distribution of EPDELNs isolated from the 11 fresh fruits and vegetables ranged from 100 to 1,000 nm, consistent with values obtained for other EPDELNs in previous studies (Ju et al., 2013; Mu et al., 2014; Wang et al., 2014).

### Small RNA sequencing results

**Figure 2:**
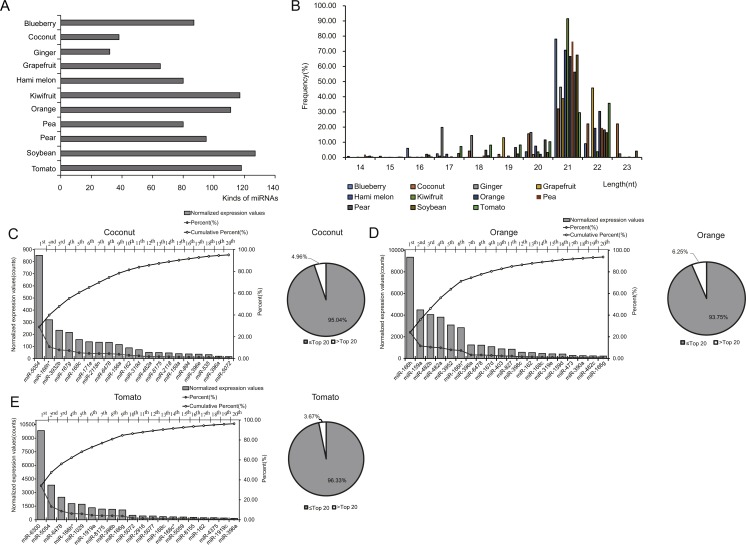
Identification and expression profiles of EPDELN-related miRNAs. (A) Number of miRNA species in different EPDELN samples. (B) Length distribution and frequency of filtered reads with matches to mature miRNAs in miRBase 21.0. (C–E) Normalized expression values and proportions (relative to all miRNAs of each EPDELN) of miRNAs in EPDELNs of coconut (C), orange (D), and tomato (E). An asterisk denotes that a miRNA with identical nomenclature is annotated in different species.

To investigate the expression profile of miRNAs in EPDELNs, total RNA of EPDELNs was extracted and subjected to capillary electrophoresis analysis. The EPDELNs were found to contain many small RNAs shorter than 25 nt in length, but little or no 18S and 28S ribosomal RNA, thus confirming the presence of small RNAs in EPDELNs (Fig. S2). Eleven small RNA libraries were then constructed (Table 1) and sequenced to generate a total of 202,560,279 raw reads. After applying a series of stringent filters, the remaining 141,076,120 reads (69.65% of raw reads) from all libraries were considered to be reliable miRNA candidates (Table 1). Because pre-miRNA sequences were unavailable in miRBase for certain edible plants and as genome sequence information was lacking for some plants in this study, the filtered reads were directly mapped to all known plant mature miRNAs in miRBase 21.0. Matching miRNAs were considered to be conserved miRNAs. In total, we identified 418 conserved miRNAs from the 11 EPDELN samples (Table S1), 32–127 per species (Fig. 2A). ELNs in ginger had the fewest miRNA species (*n* = 32) and those in soybean had the most (*n* = 127). Regarding the size distribution of the generated reads, a clear peak was observed at 20–22 nt (Fig. 2B), consistent with findings from previous studies on various plants in which the majority of small RNAs were 20–24 nt in length (Liu et al., 2015).

**Table 1:** Read abundance of miRNAs obtained by Illumina high-throughput sequencing of small RNA libraries from 11 EPDELN samples.

Species	Scientific name	Raw reads	High quality reads	High quality reads ratio (%)
Blueberry	*Vaccinium Spp*	16,913,404	14,129,331	83.54
Coconut	*Cocos nucifera*	16,983,240	13,862,158	81.62
Ginger	*Zingiber officinale*	16,050,553	8,291,742	51.66
Grapefruit	* Citrus paradisi*	23,460,112	10,293,960	43.88
Hami melon	*Cucumis melo var*	20,394,808	12,037,761	59.02
Kiwifruit	*Actinidia chinensis*	17,994,527	13,863,186	77.04
Orange	*Citrus reticulata*	16,735,652	13,319,099	79.59
Pea	*Pisum sativum*	20,101,733	13,760,883	68.46
Pear	*Pyrus spp*	18,039,882	13,954,317	77.35
Soybean	*Glycine max*	18,422,896	13,463,394	73.08
Tomato	*Solanum lycopersicum*	17,463,472	14,100,289	80.74

### Prediction of possible human target genes of plant miRNAs

**Figure 3:**
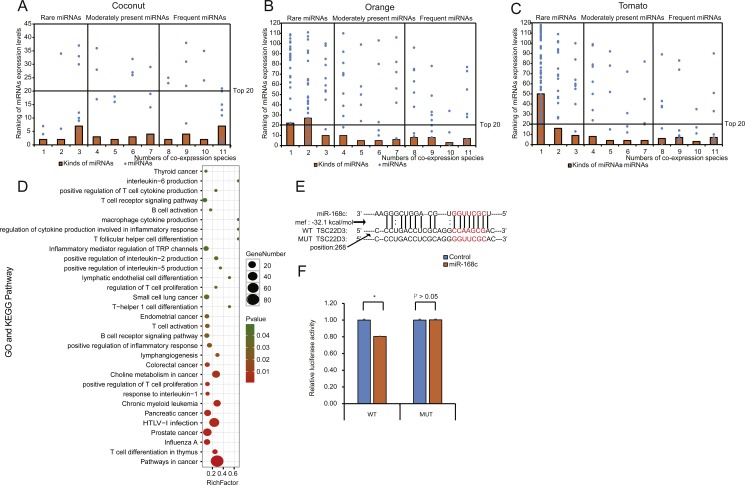
Distribution, classification, and functional analysis of miRNAs with high expression in EPDELNs. (A–C) Expression distribution of miRNAs in coconut (A), orange (B), and tomato (C). The ordinate and abscissa correspond to the ranking of miRNA expression levels and the number of co-expressed species, respectively. The terms “frequent miRNAs” (FMs), “moderately present miRNAs” (MPMs), and “rare miRNAs” (RMs) are used to describe miRNAs present almost simultaneously in 8–11, 4–7, or 1–3 EPDELN samples, respectively. The solid line is used to demarcate the top 20 expressed miRNAs of each EPDELN sample. (D) Gene Ontology and KEGG pathway analyses of categories enriched in the specific target genes of miRNAs of each EPDELN sample. The size of each circle represents the number of genes, and the color signifies the *p*-value. (E) Diagram of putative MIR-168c binding sites in TSC22D3 aligned against wild-type (WT) or mutant (MUT) MIR-168c putative target sites in the luciferase reporter plasmid. Paired bases are indicated by a black vertical line, and a mismatch is indicated by two dots. (F) Luciferase activity in HeLa cells cotransfected with MIR-168c or scrambled control oligonucleotides and the reporter constructs from (E) (*n* = 3). Statistical significance was determined by the Student’s *t*-test (*, *p* < 0.05).

miRNA expression levels in each EPDELN library varied widely, from a few RPM to thousands, with the top 20 miRNAs contributing more than 92% (92.41%–99.17%) of the total miRNA expression (Figs. 2C–2E and Fig. S3). Evidence is increasing that plant-derived miRNAs can specifically bind target mammalian mRNAs and influence biological processes (Chin et al., 2016; Zhang et al., 2012a). To further explore the potential function of EPDELN-derived miRNAs in EPDELNs, bioinformatics analysis was used to predict relationships between miRNAs and their potential target genes. We predicted the human target genes of miRNAs of each EPDELN sample (Figs. 3A–3C and Figs. S4A–S4H) using TargetScan according to the principle of base complementary pairing between the plant miRNA seed region and target genes. The results of target gene prediction suggested that some miRNAs in EPDELNs potentially target and theoretically regulate mammalian genes (Fig. 3D, Table 2, and Table S2).

**Table 2:** miRNAs and predicted target genes related inflammatory cytokine.

Species	miRNA	Sequence	Length (nt)	Predicted target gene	Gene name
Soybean	MIR-5781	UGAAACUGAGACUGCAUCUGGC	22	*IL17A*	interleukin 17A
Hami melon	MIR-164a	UGGAGAAGCAGGGCACGUGCA	21	*IL16*	interleukin 16
Orange	MIR-398b	UGUGUUCUCAGGUCGCCCCUG	21	*IL1A*	interleukin 1, alpha
Soybean	MIR-4996	UAGAAGCUCCCCAUGUUCUCA	21	*IL10*	interleukin 10
Ginger	MIR-1078	AUUGAUUCAGAUUGUGAA	18	*IL 6*	interleukin 6
Tomato	MIR-4995	GCAGUGGCUUGGUUAAGGGA	20	*IL 5*	interleukin 5
Soybean	MIR-5671a	CAUGGAAGUGAAUCGGGUGACU	22	*IL33*	interleukin 33

Recent evidence of the intake and bioavailability of dietary miRNAs in humans and animals suggests that plant-derived miRNAs possess immunomodulatory or cancer regulatory capacities (Cavalieri et al., 2016; Chin et al., 2016). Our prediction results also indicate that some of these miRNAs can directly target genes encoding inflammatory factors such as interleukin-6 (*IL-6*), interleukin-2 (*IL-2*), interleukin-5 (*IL-5*), and interleukin-1 (*IL-1*). For example, MIR-5781 in soybean ELNs can directly target *IL17A*, which plays an important role in inflammatory responses (Table 2). GO and KEGG term enrichment analyses revealed that the target genes of miRNAs in EPDELNs are related to immune cells and are significantly enriched in cancer-related signaling pathways, such as those associated with small-cell lung, endometrial, and colorectal cancers (Fig. 3D).

### EPDELN-derived miRNAs bind target genes in HeLa cells

To further validate the prediction results, we performed a dual-luciferase assay to verify the interaction between MIR-168c and its potential target gene (*TSC22D3*) (Fig. 3E and Table S3). This indicated that MIR-168c binding significantly reduced luciferase activity for the wild-type target gene, whereas binding to the mutant site had no effect on luciferase activity (Fig. 3F). MIR-8155 was similarly analyzed by dual-luciferase assay, but the result was negative (Figs. S4I, S4J and Table S3). Together, these results suggest that plant miRNAs have the potential to target mammalian mRNAs.

### Distribution of miRNA species and expression profiles across 11 EPDELN samples

**Figure 4:**
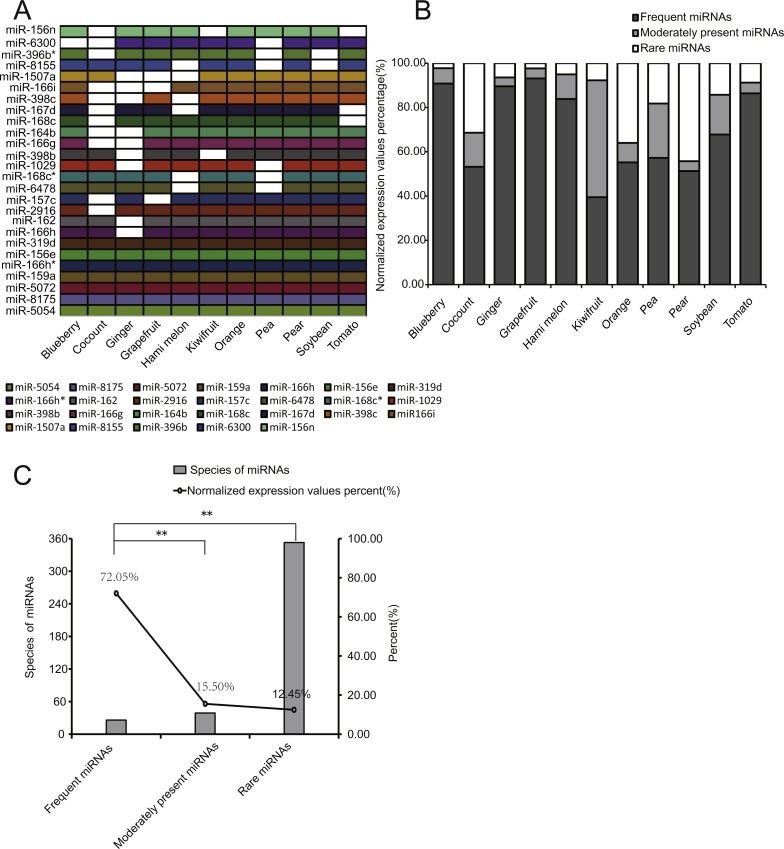
Characteristics of EPDELN-related miRNAs. (A) Expression of 26 conserved FMs in 11 EPDELN-derived miRNA libraries. The white region indicates that expression of the designated miRNA was not detected in the given ELN sample. (B) Percentage breakdown of total normalized expression of the three specific classes of miRNAs in each EPDELN sample. (C) Relationship between number of miRNA species and the proportion of normalized expression. The difference in the proportion of normalized expression between FMs and RMs was highly significant (**) according to the *t*-test.

To better compare the expression distribution of miRNAs among EPDELNs, we divided all miRNAs into three types: frequent miRNAs (FMs), moderately present miRNAs (MPMs), and rare miRNAs (RMs), corresponding respectively to miRNAs simultaneously present in 8–11, 4–7, or 1–3 kinds of EPDELNs. FMs, MPMs, and RMs comprised 26 (Fig. 4A), 39, and 353 miRNAs, respectively (Table S1).

Because miRNAs within ancient genes are more broadly and highly expressed than those within young genes (França, Vibranovski & Galante, 2016), we compared the expression levels of the three miRNA types. Notably, we found that the expression levels of FMs accounted for at least 39.46% of total miRNA expression in each EPDELN sample (Fig. 4B). This indicates that FMs have fewer miRNA species than RMs but a significantly higher cumulative expression level (Figs. 4B and 4C). Further inspection revealed that seven miRNAs were present in all 11 libraries (Fig. 4A) and were almost highly expressed miRNAs of each library (Table S1), reflecting the high expression of FMs. Among FMs, MIR-319 mainly acts on a TCP transcription factor underlying the robust and multilayered control of leaf development (Koyama, Sato & Ohmetakagi, 2017). Additionally, the overexpression of MIR-396 was shown to reduce stomatal cell number and stomatal density in *Arabidopsis* (Liu et al., 2009), and is thus of great importance for regulating plant tissue cell growth (Rodriguez et al., 2010). To screen for miRNAs highly expressed but only detected in single EPDELN samples, we identified 19 miRNAs from among the 353 RMs whose expression levels were in the top 20 of their own species (Table 3), including MIR-1919a (tomato), MIR-858a (pear), and MIR-1078 (ginger). Among these miRNAs, MIR-1078 in ginger ELNs can act on *LEP* (encoding leptin), and Fairfax et al. showed that leptin in turn associated with lipopolysaccharide-induced *IL-6* expression (Fairfax et al., 2010).

**Table 3:** The top 20 most highly expressed miRNAs unique to a particular plant ELN sample.

Species	Scientific name	miRNA	Sequence	Normalized expression values	Rank
Coconut	*Cocos nucifera*	MIR-167g	TGAAGCTGCCAGCATGATCTGA	216.16	4
		MIR-2118n	TTCCCGATGCCTCCCATTCCTA	134.62	7
Ginger	*Zingiber officinale*	MIR-1078	ATTGATTCAGATTGTGAA	2,907.68	5
		MIR-419	TGAGAATGCTGACATGAG	101.28	19
Hami melon	*Cucumis melo var*	MIR-530b	CCTGCATTTGCACCTACACCT	334.16	14
		MIR-477b	TCTCTCCCTCAAAGGCTTCTGG	400.56	12
		MIR-2111m	TAATCTGCATCCTGAGGTTT	378.67	13
		MIR-169k	TGAGCCAAGGATGACTTGCCT	267.04	15
		MIR-399d	TGCCAAAGGAGAGTTGCCCTTC	235.66	17
Kiwifruit	*Actinidia chinensis*	MIR-172a	GCGGCATCATTAAGATTCAC	265.23	14
Pear	*Pyrus spp*	MIR-858a	TTCGTTGTCTGTTCGACCT	6,502.12	2
		MIR-482a	TTCCCAAGCCCGCCCATTCCT	2,776.45	3
		MIR-1511	ACCTAGCTCTGATACCATGAA	740.25	11
		MIR-7121e	TCCTCTTGGTGATCGCCCTG	694.33	12
		MIR-479	TGTGATATTGGTTCTGGCTC	615.75	14
		MIR-482c	TCTTTCCTAACCCTCCCATTCC	374.69	16
		MIR-482b	TCTTTCCTATCCCTCCCATTCC	244.3	17
Tomato	*Solanum lycopersicum*	MIR-1919a	ACGAGAGTCATCTGTGACAG	1,327.08	6
		MIR-1919c	TGTCGCAGATGACTTTCGCCC	194.64	19

## Discussion

Previous studies have indicated that the size and structure of exosome-like nanoparticles in edible plants are similar to those of mammalian-derived exosomes (Ju et al., 2013; Mu et al., 2014; Zhang et al., 2016). Our findings based on AFM and DLS analyses support the viewpoint of these earlier studies. In our present study, we found that EPDELNs have two kinds of distributions, one up to 100 nm and the other up to 1,000 nm, which is consistent with previous results (Mu et al., 2014; Zhuang et al., 2015). Interestingly, Zhuang et al. (2015) found that these two types of ELNs have differences in composition and absorption patterns.

As expected, we detected large numbers of miRNAs in plant-derived ELNs (Mu et al., 2014). Consistent with other studies (He et al., 2015), we found commonalities and differences in miRNA species and expression levels in different plants. miRNAs are involved in diverse aspects of plant growth and development (Mallory & Vaucheret, 2006; Sunkar et al., 2007). Some of the 26 miRNAs that were always present in 8–11 ELNs according to our bioinformatics analysis were shown to participate in fundamental plant physiological and biochemical processes, such as various stress responses, flowering regulation, sugar accumulation, and root development (Llave, 2004; Dong et al., 2009; Khan et al., 2011; Kim et al., 2014; Sunkar & Zhu, 2004; Yu et al., 2015; Zhao et al., 2017). For example, the MIR-319 family acts on *TCP*, forming a relationship that has implications for many aspects of floral development (Nag et al., 2009). MIR-398 also participates in plant responses to biotic, heavy metal, high-salt, drought, and UV radiation stresses by targeting two superoxide dismutase genes (*CSD1* and *CSD2*) (Jagadeeswaran & Sunkar, 2009; Lu et al., 2011). An auxin response factor was predicted to be a target of MIR-167 (Yu et al., 2015), and these DNA-binding proteins are thought to control transcription in response to the phytohormone auxin (Hagen & Guilfoyle, 2002; Yu et al., 2015). FMs detected in the present study included the above-mentioned miRNAs. Some identified miRNAs may be associated with biological diversity, in other words, related to plant-specific phenotypes. Anthocyanins, which play a role in the change of color of ripening fruit (Kayesh et al., 2013), are found in most other plant parts and in most plant genera (Zhang & Furusaki, 1999). Anthocyanin pigments may be red, purple, or blue, depending on the pH (Araceli et al., 2009). The overexpression of MIR-156e from herbaceous peony improves anthocyanin accumulation in transgenic *Arabidopsis* (Zhao et al., 2017). In our present study, MIR-156e expression levels differed among the 11 EPDELN samples. The regulation of plant anthocyanins by miRNAs is probably at least partially responsible for the widespread variation in plant coloration.

Recent studies have shown that plant-derived ELNs can retain their stability and be absorbed through the GI tract (Ju et al., 2013; Mu et al., 2014; Raimondo et al., 2015; Wang et al., 2014). Additionally, functional studies have suggested that plant-derived ELNs play a role in interspecies intercellular communication and function against inflammatory diseases (Ju et al., 2013) and cancers (Raimondo et al., 2015). Our results indicate that miRNAs are abundant in EPDELNs, while some of them have been reported to be involved in cross-species regulation. For example, *Moringa oleifera*-derived MIR-166i functions in the regulation of inflammation (Stefano et al., 2016), while honeysuckle-derived MIR-2911 inhibits influenza A viruses (Zhen et al., 2015). Moreover, rice-derived MIR-168a specifically targets and regulates *LDLRAP1* expression in mouse livers (Zhang et al., 2012a). In this study, our GO and KEGG analyses of putative target genes of highly expressed plant miRNAs revealed enriched pathways associated with immunity and cancer. Chin et al. (2016) recently reported that long-term oral intake of plant MIR-159 can suppress breast tumor growth by targeting *TCF7* which encodes a Wnt signaling transcription factor, leading to a decrease in MYC protein levels. Takagi et al. (2011) found that the target gene (*TSC22D3)* of plant MIR-168c in non-tumorous tissue of remnant liver was significantly associated with early recurrence of hepatocellular carcinoma (HCC) after surgical resection. Moreover, our dual-luciferase reporter system analysis demonstrated that plant MIR-168c in ELNs binds *in vitro* to the potential target gene *TSC22D3*, suggesting that MIR-168c has the potential to be used after surgical resection of HCC. Taken together, our results and those of previous studies imply that miRNAs in ELNs are crucial factors underlying ELN regulatory functions.

Several research groups recently demonstrated that artificially synthesized nanoparticles can be used to target low doses of drugs (e.g., small interfering RNAs, proteins, and peptides) to specific cell types and tissues (Wilson et al., 2010; Xiao et al., 2015a; Xiao et al., 2014; Xiao et al., 2015b). From a therapeutic perspective, plant-derived edible nanoparticles are safe and more amenable to mass production than synthetic nanoparticles (Zhang et al., 2016). They may therefore be used as efficient nanofactors for the fabrication of specific drugs deliverable as medical nanoparticles, which is a potentially novel approach to nanomedicine (Ju et al., 2013). However, a detailed elucidation of the molecular mechanism underlying the uptake and action of plant-derived miRNAs in EPDELNs is still urgently needed.

## Conclusions

In this study, we analyzed the miRNA profiles of EPDELNs of 11 different fruits and vegetables. Although plant-derived ELNs contain many miRNAs, the types and levels of miRNAs differ markedly among species. We identified 418 miRNAs with varying EPDELN distributions. FMs were represented by fewer miRNA species than RMs but had a significantly higher cumulative expression level. Highly expressed miRNAs in EPDELNs can potentially regulate the expression of inflammatory cytokines and cancer-related genes *in vitro*.

##  Supplemental Information

10.7717/peerj.5186/supp-1 Figure S1 EPDELNs morphological ultrastructure and size distribution The morphological ultrastructure was visualized by AFM, and the size distribution of EPDELNs was analyzed by DLS in blueberry (A); ginger (B); grapefruit (C); kiwifruit (D); orange (E); pea (F); pear (G); soybean (H). Photographs by Juan Xiao.

10.7717/peerj.5186/supp-2 Figure S2 Identification of miRNAs in ELNs of edible plants RNA extracted from EPDELNs was detected using Agilent 2100 Bioanalyzer. Blueberry (A); coconut (B); ginger (C); grapefruit (D); Hami melon(E); kiwifruit (F); orange (G); pea (H); pear (I); soybean (J); Tomato (K).

10.7717/peerj.5186/supp-3 Figure S3 EPDELNs-related miRNA expression Profiles Normalized expression values and proportions (relative to all miRNAs of each EPDELN) of the top 20 miRNAs in EPDELNs of blueberry(A); ginger (B); grapefruit (C); Hami melon (D); kiwifruit (E); pea (F); pear (G); soybean (H). An asterisk denotes that a miRNA with identical nomenclature is annotated in different species.

10.7717/peerj.5186/supp-4 Figure S4 Characteristics of EPDELN-related miRNAs Expression distribution of miRNAs in blueberry (A); ginger (B); grapefruit (C); Hami melon (D); kiwifruit (E); pea (F); pear (G); soybean (H). The ordinate and abscissa correspond to the ranking of miRNA expression levels and the number of co-expressed species, respectively. The terms “frequent miRNAs” (FMs), “moderately present miRNAs” (MPMs), and “rare miRNAs” (RMs) are used to describe miRNAs present almost simultaneously in 8–11, 4–7, or 1–3 EPDELN samples, respectively. The solid line is used to demarcate the top 20 expressed miRNAs of each EPDELN sample. (I) Diagram of the putative MIR-8155 binding sites in IL8, and luciferase reporter plasmid containing the wild-type (WT) or mutant (MUT) MIR-8155 putative target site. Paired bases were indicated by a black vertical and mispairing was indicated by two dots. (J) Luciferase activities in Hela cells co-transfected with MIR-8155 or scrambled control oligos and the reporter constructs from I. (*n* = 3). Statistical significant was determined by Student’s *t*-test (* *P* < 0.05).

10.7717/peerj.5186/supp-5 Table S1 Merged normalized expression and sequenceuence of all identification miRNAs in 11 species All miRNAs are named MIR- based on the plant miRNA maturation nomenclature.

10.7717/peerj.5186/supp-6 Table S2 Go and KEGG data of high expression of top 20 miRNAs

10.7717/peerj.5186/supp-7 Table S3 List of the Homo sapiens potential target genes predicted for miR-168c or miR-8155

## Abbreviations

miRNA: microRNA
EPDELNs: Edible plant-derived exosome-like nanoparticles
FMs: Frequent miRNAs
MPMs: Moderately present miRNAs
RMs: Rare miRNAs
ELNs: Exosome-like nanoparticles
LDLRAP1: Lipoprotein receptor adapter protein 1
AFM: Atomic force microscopy
DLS: Dynamic light scattering
*IL-6*: interleukin-6
*IL-2*: interleukin-2
*IL-5*: interleukin-5
*IL-1*: interleukin-1
GO: Gene Ontology
KEGG: Kyoto Encyclopedia of Genes and Genomes
WT: Wild type
MUT: Mutant

## Additional Information and Declarations

### Data Availability

The following information was supplied regarding data availability: GEO: https://www.ncbi.nlm.nih.gov/geo/query/acc.cgi?acc=GSE116095.
