# Supplementary material for: Identification of exosome-like nanoparticle-derived microRNAs from 11 edible fruits and vegetables
Source: PeerJ. 2018 Jul 31;6:e5186. doi: 10.7717/peerj.5186 (PMC6074755; doi:10.7717/peerj.5186)
Supplement: Figure S1 — The morphological ultrastructure was visualized by AFM, and the size distribution of EPDELNs was analyzed by DLS in blueberry (A); ginger (B); grapefruit (C); kiwifruit (D); orange (E); pea (F); pear (G); soybean (H). Photographs by Juan Xiao. [file peerj-06-5186-s001.pdf]

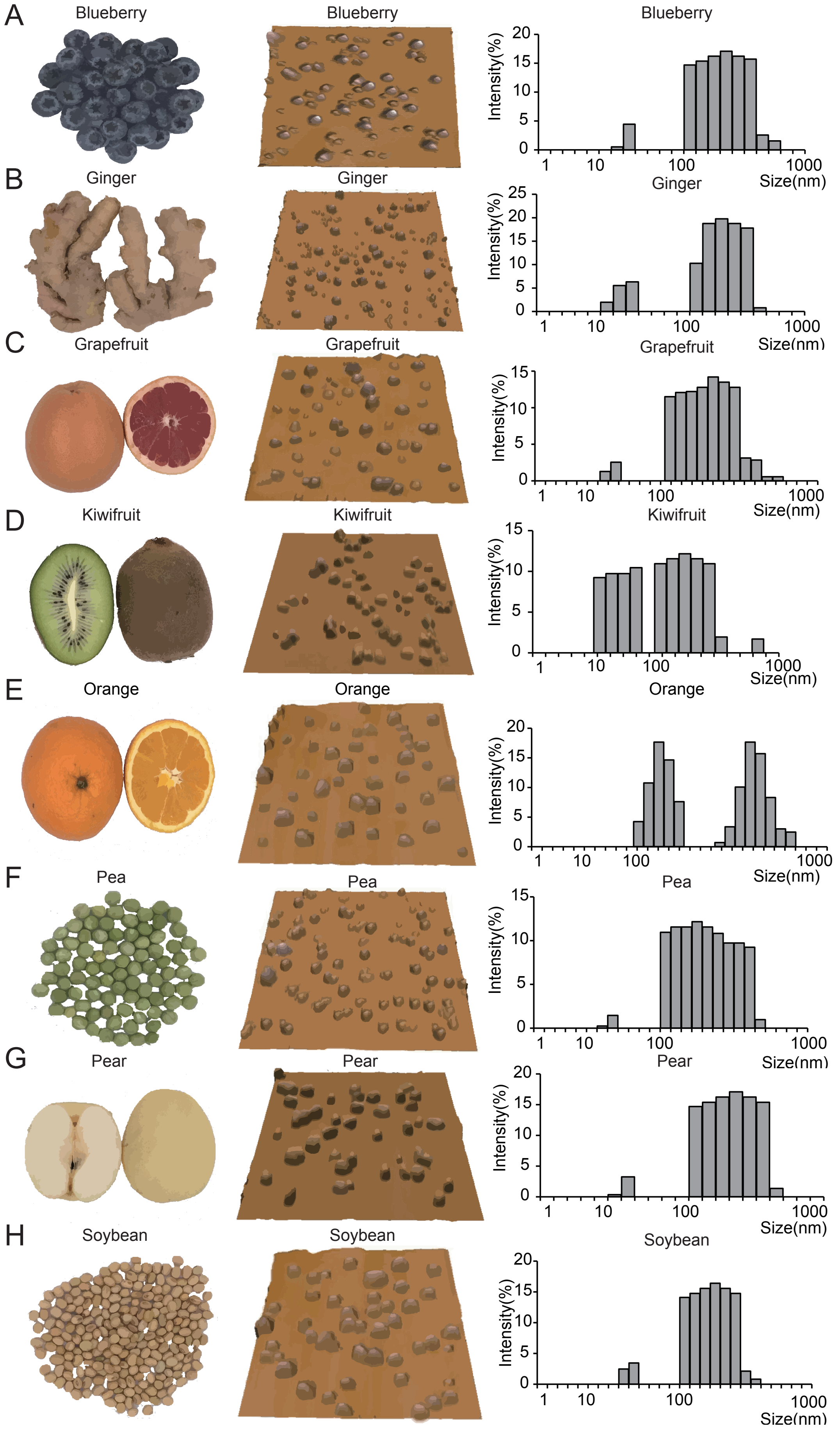
A
Blueberry
Blueberry
Blueberry
Intensity(%)
Size(nm)
B
Ginger
Ginger
Ginger
Intensity(%)
Size(nm)
C
Grapefruit
Grapefruit
Grapefruit
Intensity(%)
Size(nm)
D
Kiwifruit
Kiwifruit
Kiwifruit
Intensity(%)
Size(nm)
E
Orange
Orange
Orange
Intensity(%)
Size(nm)
F
Pea
Pea
Pea
Intensity(%)
Size(nm)
G
Pear
Pear
Pear
Intensity(%)
Size(nm)
H
Soybean
Soybean
Soybean
Intensity(%)
Size(nm)
