# Supplementary figures and images for: Identification of exosome-like nanoparticle-derived microRNAs from 11 edible fruits and vegetables
Source: PeerJ. 2018 Jul 31;6:e5186. doi: 10.7717/peerj.5186 (PMC6074755; doi:10.7717/peerj.5186)

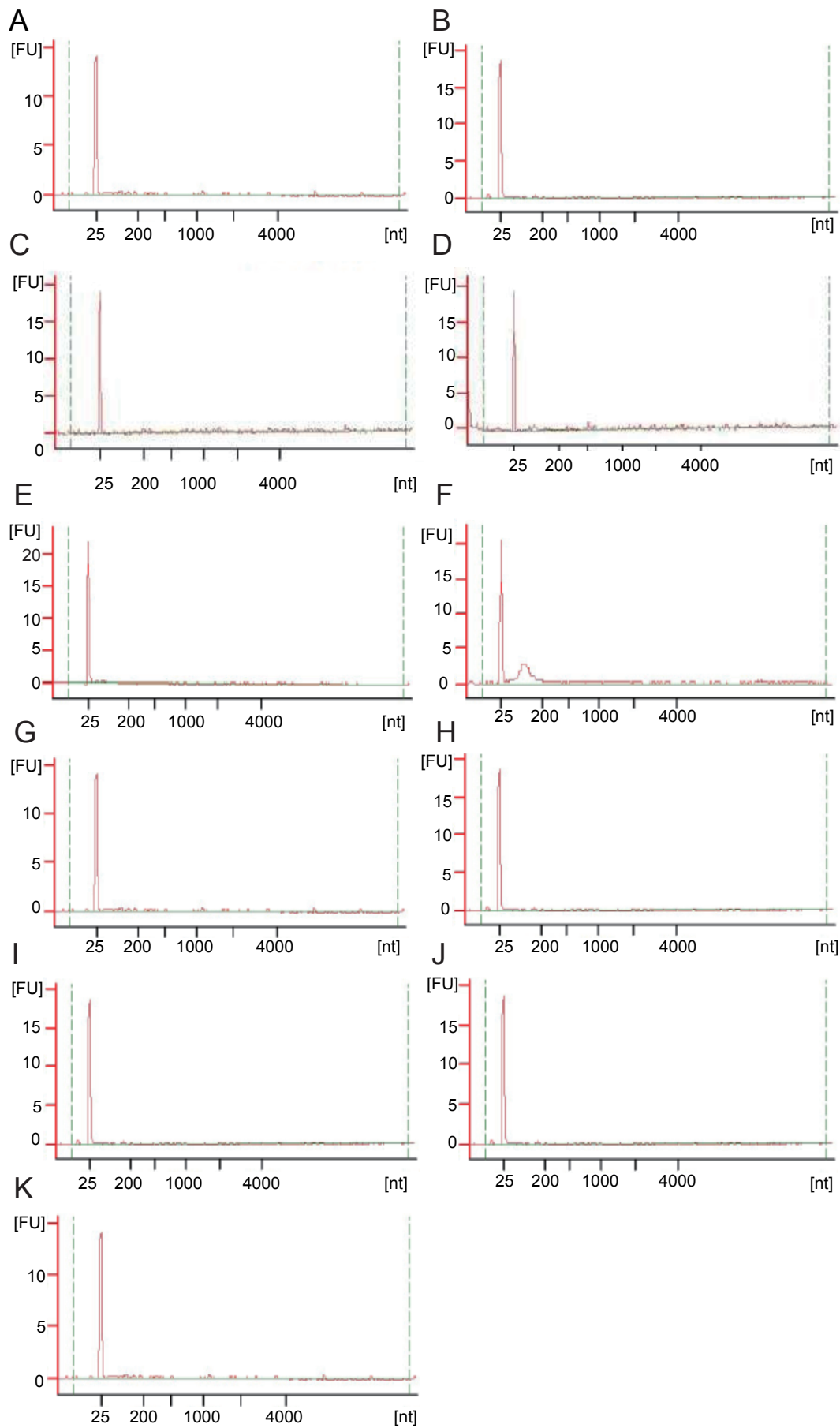

Supplement: Figure S2 — RNA extracted from EPDELNs was detected using Agilent 2100 Bioanalyzer. Blueberry (A); coconut (B); ginger (C); grapefruit (D); Hami melon(E); kiwifruit (F); orange (G); pea (H); pear (I); soybean (J); Tomato (K). [file peerj-06-5186-s002.pdf]

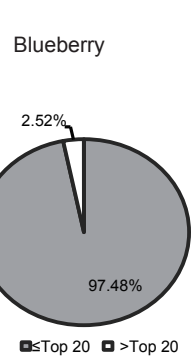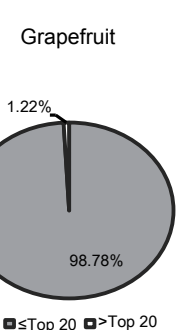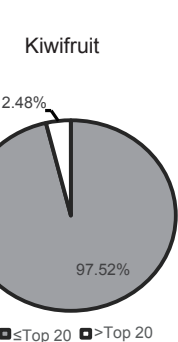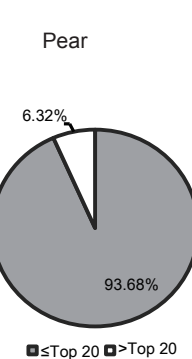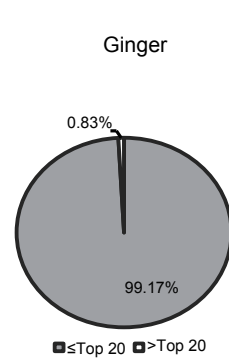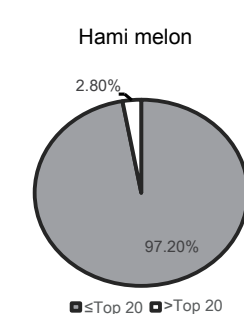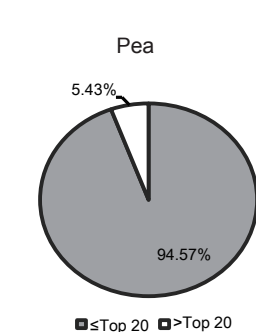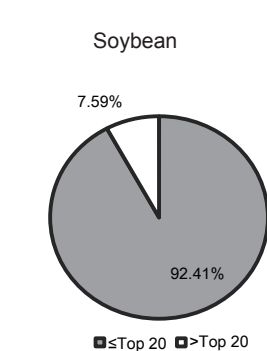

Supplement: Figure S3 — Normalized expression values and proportions (relative to all miRNAs of each EPDELN) of the top 20 miRNAs in EPDELNs of blueberry(A); ginger (B); grapefruit (C); Hami melon (D); kiwifruit (E); pea (F); pear (G); soybean (H). An asterisk denotes that a miRNA with identical nomenclature is annotated in different species. [file peerj-06-5186-s003.pdf]

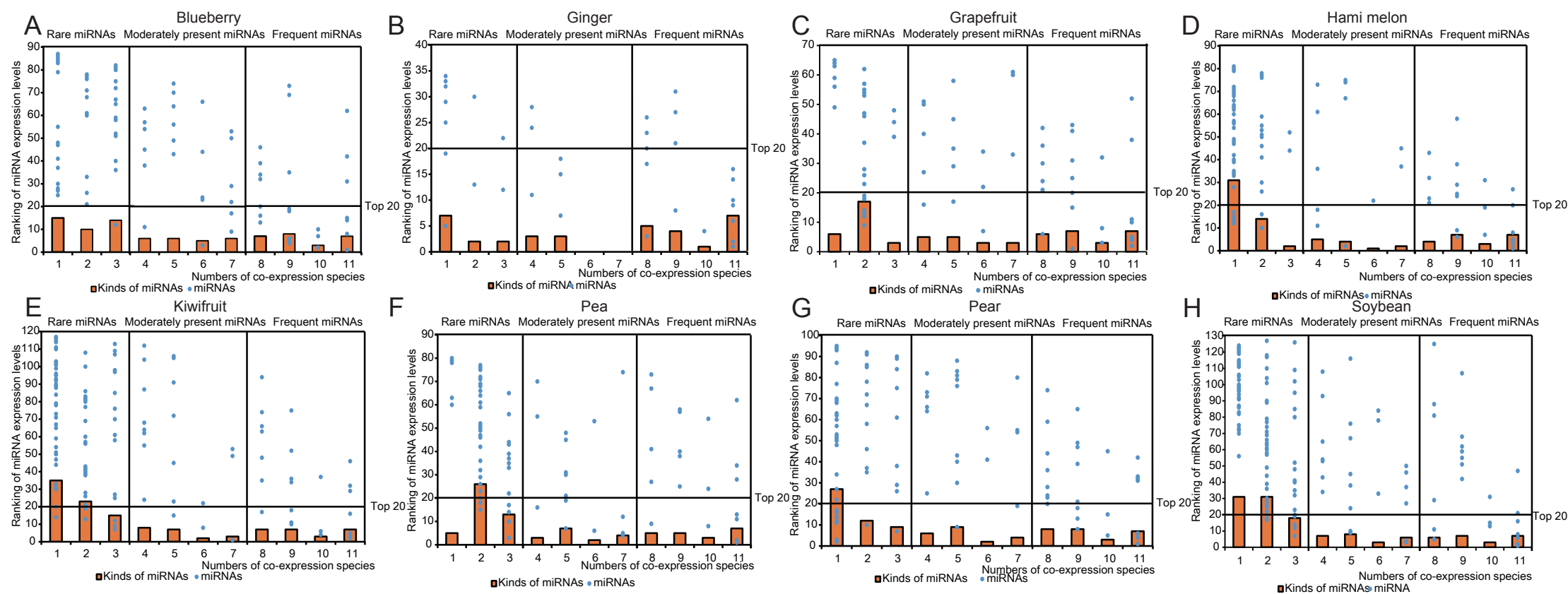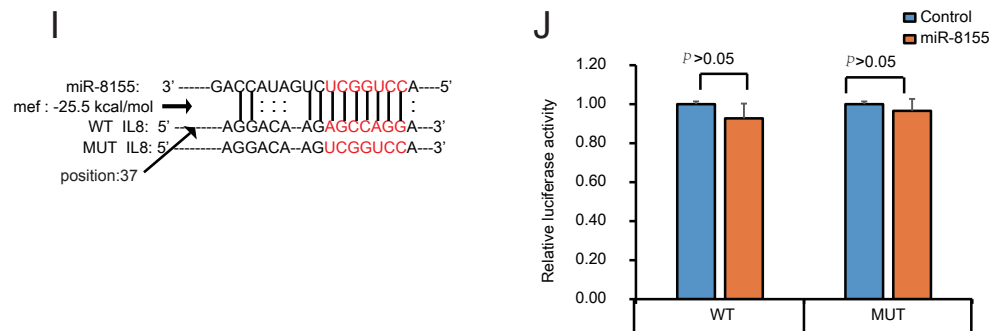

Supplement: Figure S4 — Expression distribution of miRNAs in blueberry (A); ginger (B); grapefruit (C); Hami melon (D); kiwifruit (E); pea (F); pear (G); soybean (H). The ordinate and abscissa correspond to the ranking of miRNA expression levels and the number of co-expressed species, respectively. The terms “frequent miRNAs” (FMs), “moderately present miRNAs” (MPMs), and “rare miRNAs” (RMs) are used to describe miRNAs present almost simultaneously in 8–11, 4–7, or 1–3 EPDELN samples, respectively. The solid line is used to demarcate the top 20 expressed miRNAs of each EPDELN sample. (I) Diagram of the putative MIR-8155 binding sites in IL8, and luciferase reporter plasmid containing the wild-type (WT) or mutant (MUT) MIR-8155 putative target site. Paired bases were indicated by a black vertical and mispairing was indicated by two dots. (J) Luciferase activities in Hela cells co-transfected with MIR-8155 or scrambled control oligos and the reporter constructs from I. (n = 3). Statistical significant was determined by Student’s t-test (* P < 0.05). [file peerj-06-5186-s004.pdf]
