# Supplementary material for: Identification of exosome-like nanoparticle-derived microRNAs from 11 edible fruits and vegetables
Source: PeerJ. 2018 Jul 31;6:e5186. doi: 10.7717/peerj.5186 (PMC6074755; doi:10.7717/peerj.5186)
Supplement: Table S2 [file peerj-06-5186-s006.docx]

Supplementary Table 2. Go and KEGG data of high expression of top 20 miRNAs

| Number | Term | Genes | *P*-Value |
| --- | --- | --- | --- |
| bta05200 | Pathways in cancer | 87 | 3.76E-09 |
| GO:0033077 | T cell differentiation in thymus | 6 | 1.49E-03 |
| cfa05164 | Influenza A | 11 | 1.61E-03 |
| bta05215 | Prostate cancer | 28 | 2.29E-03 |
| cfa05166 | HTLV-I infection | 45 | 3.02E-03 |
| bta05212 | Pancreatic cancer | 15 | 3.61E-03 |
| cfa05220 | Chronic myeloid leukemia | 18 | 3.68E-03 |
| GO:0070555 | response to interleukin-1 | 3 | 5.50E-03 |
| GO:0042102 | positive regulation of T cell proliferation | 4 | 6.24E-03 |
| cfa05231 | Choline metabolism in cancer | 23 | 7.19E-03 |
| cfa05210 | Colorectal cancer | 5 | 9.19E-03 |
| GO:0001946 | lymphangiogenesis | 3 | 1.69E-02 |
| GO:0050729 | positive regulation of inflammatory response | 5 | 1.91E-02 |
| ptr04662 | B cell receptor signaling pathway | 5 | 2.48E-02 |
| GO:0042110 | T cell activation | 4 | 2.67E-02 |
| cfa05213 | Endometrial cancer | 4 | 2.75E-02 |
| GO:0045063 | T-helper 1 cell differentiation | 2 | 3.17E-02 |
| ptr05222 | Small cell lung cancer | 6 | 3.38E-02 |
| GO:0042129 | regulation of T cell proliferation | 2 | 3.57E-02 |
| GO:0060836 | lymphatic endothelial cell differentiation | 2 | 3.85E-02 |
| GO:0032754 | positive regulation of interleukin-5 production | 2 | 3.90E-02 |
| GO:0032743 | positive regulation of interleukin-2 production | 3 | 4.06E-02 |
| cfa04750 | Inflammatory mediator regulation of TRP channels | 6 | 4.27E-02 |
| GO:0061470 | T follicular helper cell differentiation | 2 | 4.48E-02 |
| GO:1900015 | regulation of cytokine production involved in inflammatory response | 2 | 4.56E-02 |
| GO:0010934 | macrophage cytokine production | 2 | 4.56E-02 |
| GO:0042113 | B cell activation | 2 | 4.59E-02 |
| GO:0050852 | T cell receptor signaling pathway | 4 | 4.62E-02 |
| GO:0002726 | positive regulation of T cell cytokine production | 2 | 4.68E-02 |
| GO:0032758 | interleukin-6 production | 2 | 4.74E-02 |
| cfa05216 | Thyroid cancer | 2 | 4.89E-02 |
