# Supplementary material for: Identification of exosome-like nanoparticle-derived microRNAs from 11 edible fruits and vegetables
Source: PeerJ. 2018 Jul 31;6:e5186. doi: 10.7717/peerj.5186 (PMC6074755; doi:10.7717/peerj.5186)
Supplement: Table S3 [file peerj-06-5186-s007.docx]

Supplementary Table 3 List of the Homo sapiens potential target genes predicted for miR-168c or miR-8155

| miRNA name | sequence | Target NCBI Accession | Target gene name | Target Region | mfe (kcal/mol) |
| --- | --- | --- | --- | --- | --- |
| MIR-168c | UCGCUUGGUGCAGGUCGGGAA | XM_005262103.3 | *TSC22D3* | CDS | -39.1 |
| MIR-8155 | ACCUGGCUCUGAUACCAG | AK311874.1 | *IL8* | 3' UTR | -25.5 |
